# Two decades of safe abortion policy evolution and reform in Nepal (2002–2025): progress, implementation, challenges, and opportunities

**DOI:** 10.1186/s13690-026-01949-5

**Published:** 2026-05-20

**Authors:** Geha Nath Khanal, Resham B Khatri

**Affiliations:** 1 Nepal Public Health Association, Lalitpur, Nepal; 2 School of School of Nursing, Midwifery, Allied and Public Health, Canterbury Christ Church University, Canterbury, UK; 3 School of Public Health, University of Queensland, Brisbane, Australia; 4 Health Social Science and Development Research Institute, Kathmandu, Nepal

**Keywords:** Safe abortion, Reproductive rights, Policy analysis, Implementation challenges, Nepal

## Abstract

**Background:**

Nepal has made significant strides in reproductive rights, legalizing safe abortion services (SAS) in 2002, and recognizing them as fundamental rights in its 2015 constitution. Despite this, over half of abortions remain unsafe, with persistent disparities in access to and utilization of safe abortion services. This study examines the trajectory of Nepal’s abortion policy evolution and reform process, and implementation status, and ongoing challenges and opportunities using the heuristic policy cycle framework to inform evidence-based strategies for equitable reproductive rights.

**Methods:**

A policy process and content analysis was conducted using a mixed-methods approach. To identify relevant policy documents and literature, we used search terms related to three concepts: abortion, law, and location (Nepal). We identified 25 peer-reviewed studies and 32 grey literature policy documents published in English or Nepali up to 30 June 2025, identified via PubMed, Embase, Medline Ovid, Google Scholar, and government sources. Quantitative data were extracted from government annual reports and national census data. Data analysis involved thematic coding of qualitative data across five policy stages (agenda setting, formulation, adoption, implementation, and evaluation) and descriptive analysis of quantitative service utilization and the sex ratio at birth (SRB) data.

**Results:**

Agenda setting of the legalization of abortion in Nepal was driven by high maternal mortality ratio and advocacy aligning with global commitments. The 2002 legal reform legalized abortion up to 12 weeks on demand, with provision requiring spousal consent, and banned on sex-selective abortion. Implementation efforts focused on capacity building, service expansion—expanding SAS to 1,685 sites—and equitable distribution of services, delivering 1.6 million SAS over two decades. The proportion of medical abortion has increased from 50% to 73% of cases. However, 53% of pregnancies are unintended, with abortion often used as contraception. More than 50% of service providers are outside the formal system. There is inequitable access to SAS, and the SRB is skewed towards male, indicating ongoing sex-selective abortion.

**Conclusions:**

Nepal’s abortion policy journey represents remarkable progress in legal reforms and service expansion; however several contextual and systemic barriers such as low awareness, rural inequity, unregulated providers, sex selective abortion, and the repeated use of abortion as family planning undermine equitable access and reproductive rights. Key reforms include expanding second-trimester services, strengthening regulations, combating sex-selective abortion practices, enhancing access to family planning services, and implementing abortion related awareness campaigns to address gender discrimination, service information gaps, and unintended consequences of unsafe practices.

**Table:** 

Text box 1: Contributions to the literature
• Provides the first comprehensive mixed-methods analysis of Nepal’s safe abortion policy (2002–2025).
• Identifies critical gaps between progressive legislation and implementation, including rural–urban disparities, dependence on unregulated providers, use of abortion as means of family planning, and persistently skewed sex ratio at birth
• Theoretically, it validates the policy cycle framework for examining health policy in low- and middle-income countries.
• Methodologically, it integrates qualitative policy review with quantitative evidence.
• Offers practical, evidence-based, and actionable recommendations to expand equitable access and strengthen the realization of reproductive rights.

## Background

Globally, approximately 73 million abortions take place annually, 45% of which remain unsafe [1]. These unsafe abortions are predominantly concentrated in low and middle-income countries (LMICs), which account for about 97% of these cases [2], with 50% occurring in Asia alone [3–5]. Unsafe abortions contribute to 5–13% of maternal deaths and cause severe complications including post-abortion sepsis, haemorrhage, genital trauma, infection, and infertility [1, 2]. In response, the World Health Organization (WHO) has focused on eliminating unsafe abortions through legal reforms, strengthening the health system and community interventions for equitable access to Safe Abortion Services (SAS) [1, 2, 6]. Currently, 60% of women of reproductive age live in countries where abortion laws are broadly legal; while remaining 40% reside in regions with restrictive laws, allowing abortion only in cases where the women’s health or life is at risk, rape, incest, or fetal abnormalities. Out of these women, 6% live in the 21 countries where abortion is completely banned [7].

In the last three decades, more than 60 countries have liberalized their abortion laws, making a shift towards reproductive freedom [7]. This liberalization aims to improve maternal health outcomes and recognize reproductive health rights [2]. In contrast, four countries; Poland, the United States, Nicaragua, and El Salvador; have rolled back abortion rights due to various reasons [7]. In the United States, this shift was ideologically driven, exemplified by the ruling by the US supreme court in 2022 that overturned the landmark *Roe v. Wade* decision of 1973 [8]. Similarly, in Poland, restrictive abortion laws have been shaped by Catholic pro-life teachings [9]; while in Nicaragua and El Salvador, conservative political and religious ideologies have reinforced criminalization [10, 11]. These reversals increase the risks of unsafe abortions, undermining the global maternal health initiatives.

Nepal’s journey toward liberalizing abortion has been shaped by deeply rooted cultural and legal barriers. Abortion was traditionally viewed as immoral and strictly forbidden, stemming from long-standing social norms that criminalized the act and imposed severe punishments for women [12, 13]. The Country Code of 1854, and its revision in1963, prohibited abortion except to save the women’s life [13]. Women seeking abortion faced 1–3 years’ imprisonment, and for abortions after 28 weeks’ gestation, they could be charged with infanticide, facing up to 20 years’ imprisonment [13, 14].

A major legal reform in 2002 marked a turning point in Nepal, when abortion was legalized on demand up to 12 weeks and up to 18 weeks in case of rape or incest, and anytime if the pregnancy posed a threat to a women’s physical or mental health or fetal abnormality [13, 15].The impetus for the 2002 legal reform was due to the combination of public health crisis and sustained human rights advocacy. This change was operationalized in public health service delivery in 2004 through initiation of comprehensive abortion care (CAC) services, initially focusing on first trimester abortion [16]. The scope was further expanded in 2007 to include second-trimester pregnancies. Later in 2009, medical abortion (MA) was introduced, which dramatically increased safe and accessible services for women, including those in remote areas [17–19]. By 2023, over 1.6 million women had accessed SAS, reflecting a significant progress in women’s rights as well as aligning national policies with international commitments to combat unsafe abortion [20].

Although a substantial number of research on safe abortion in Nepal has been conducted, the existing literatures have focussed on service utilization and outcomes [3, 21], factors associated with unsafe abortion [22–24], the impact of legalization [18], barriers and facilitators using SAS [18, 25, 26], women’s experiences with services [18, 27, 28], knowledge of abortion laws [29, 30], and provider perspectives [4, 31]. Some studies have also examined the policy development process and implementation challenges [12, 17], including the effect of policy changes to increased access and coverage following federalism [32].

However, a critical gap remains: there is no comprehensive analysis of the entire policy process; right from agenda setting that led to 2002 reforms, to policy implementation and evaluation. A comprehensive analysis of Nepal’s abortion policy journey can reveal how the agenda came under consideration, what strategies were adopted to implement the policy, what bottlenecks emerged during the implementation, thereby offering evidence-based insights not only for strengthening Nepal’s own policy but also for informing policy and advocacy in other LMICs initiating similar reforms.

This study aims to analyze each stage of the policy cycle to provide evidence-based insights for policymakers and stakeholders, informing future reforms, enhancing access to SAS, and advancing equitable reproductive rights in Nepal.

## Methods

We conducted a policy analysis to examine the development process of Nepal’s safe abortion policy, tracing its evolution from criminalization to the recognition of abortion as fundamental constitutional right. We adopted a mixed-methods review approach, integrating qualitative and quantitative data to explain key stages and challenges in the safe abortion policy development process. Qualitative data were extracted from a comprehensive review of policy documents, legal documents and frameworks, peer-reviewed literature, and newspaper and magazine reports, while quantitative data were extracted from annual reports on service utilization and demographic composition. This mixed-methods approach provided a comprehensive understanding of the policy’s impact.

### Data sources

This study collected documents through a comprehensive but non-systematic literature search using an iterative approach. We searched three main databases (PubMed, Embase and Medline Ovid) and Google Scholar to find additional studies. Our search strategy employed keywords in three categories: (a) Abortion (Abortion, Safe Abortion, Pregnancy Termination); (b) law (Law, Legal, Legalization, Criminalization, Decriminalization); and (c) Location (Nepal, Nepalese). We used Boolean operators (AND, OR) along with truncations (*) to each database’s requirements. We included English-language, full-text publications up to 30 June 2025, to capture the evolution of abortion policy reforms.

We also looked for grey literature in both English and Nepali by searching official governmental sources. We reviewed the documents like constitution, laws, regulations, policies, guidelines, annual reports, circulars, service tracking dashboard, and newspaper articles. We checked the website of key governmental institutions like Federal Parliament, Ministry of Health and Population (MoHP), Department of Health Services (DoHS), Family Welfare Division, and National Statistics Office to identify these relevant documents. For news coverage and reporting, we searched Google news using the same keywords from our academic search focusing on articles published between 2021 and 2025 and limiting it to the first 100 results. Finally, we scanned the reference list of the documents that we had already gathered to track any other relevant grey literature that might have missed.

For quantitative data, we extracted abortion service utilization data from annual reports of DoHS from 2003/04 to 2023/24 [16, 20, 33–40]. To look at the sex ratio at birth, we used the data form Nepal’s population census along with the information from other relevant journal articles [41, 42]. Any documents that were not relevant or did not fit within the scope of the policy development cycle were excluded from the analysis.

### Data analysis and synthesis

We developed a data charting form to systematically extract relevant information from each study, including author, year, document type, key concepts, and main findings. The first author conducted data extraction, while the second author double-checked it. Whenever there were any discrepancies, the team resolved through consensus.

For peer-reviewed articles, we used Zotero—a reference management tool—to manage the data [43]. We analysed and mapped the extracted data using policy cycle framework, which breaks policymaking into five stages: problem identification and agenda setting, policy formulation, policy decision-making and adoption, implementation, and evaluation [44]. We analysed and interpreted the textual data based on their relevance to the policy development cycle using both deductive and inductive approaches. Themes and codes were aligned with the heuristic model of public policymaking.

For quantitative analysis, we extracted the number and types of SAS used over the period and sex ratio at birth data. We pulled the data and compiled in a spreadsheet, and used the program R (version 4.4.3) for making visual graphs [45].

## Results

**Fig. 1:**
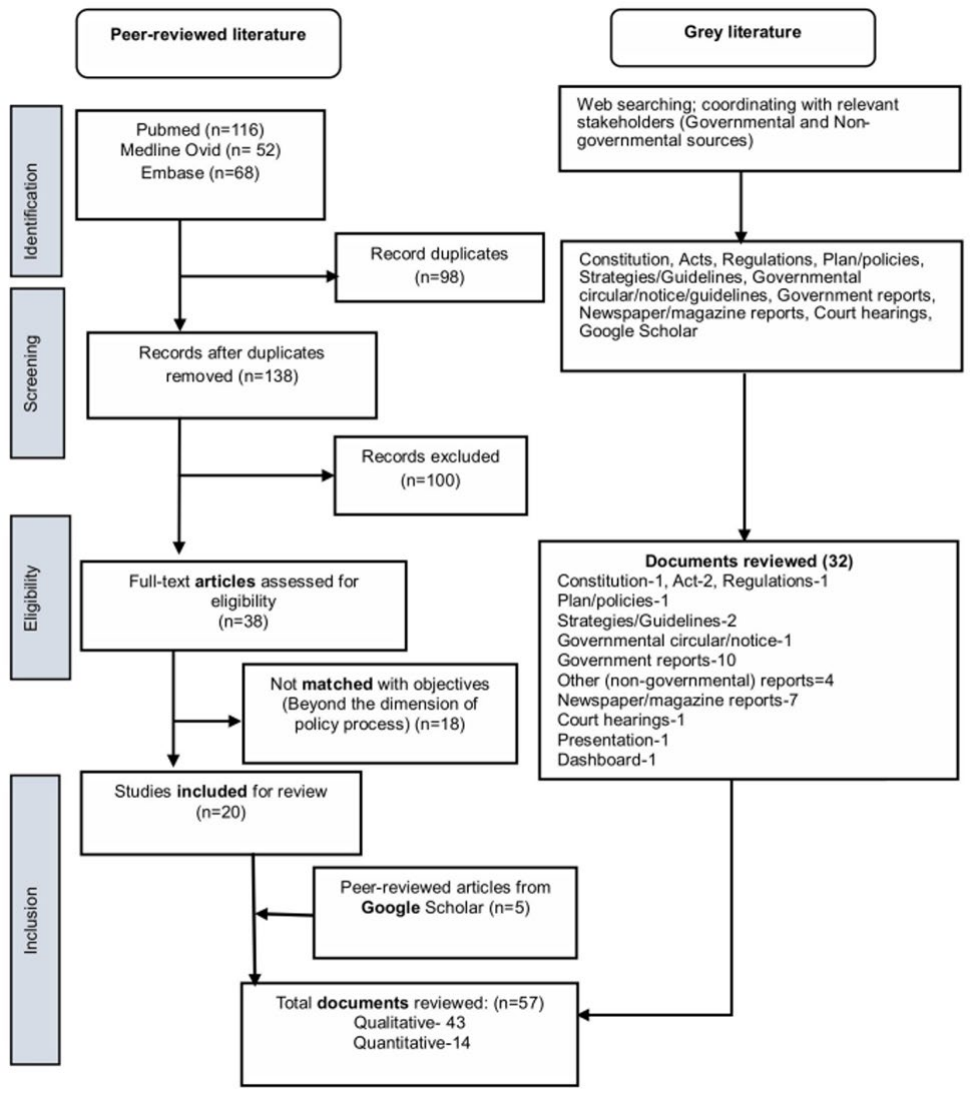
Flow chart showing the selection of studies for this study

The database search identified 236 articles. After removing duplicates, 138 unique articles remained. After screening titles and abstracts, 100 articles were excluded, leaving 38 articles for full-text eligibility assessment. Following full-text review, 20 articles were included in the final review. Additionally, 32 grey literature sources and 5 articles from Google Scholar were included, resulting in a total of 57 data sources in the final review (Fig. 1).

These studies were published between 1997 and 2025 and covered a wide range of document type. There were peer-reviewed articles (44%), policy documents (constitution, acts, plans/policies, strategies/guidelines, circulars/notices) (12%), reports/dashboards (28%), newspaper/magazine articles (12%), and court hearings and presentations (4%).

**Fig. 2:**
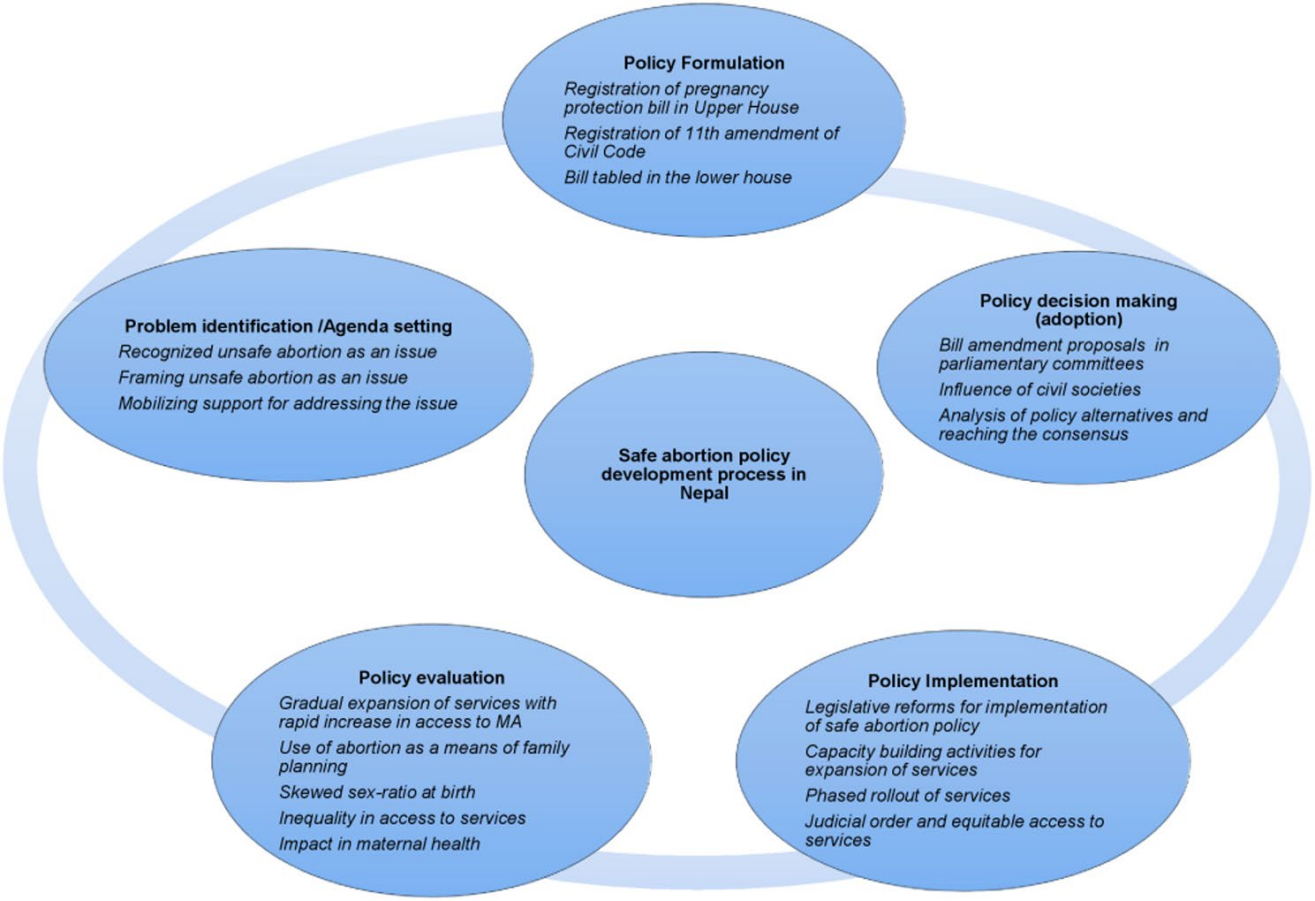
Five step process of safe abortion policy development in Nepal

Figure 2 illustrates the SAS policy development process in Nepal, highlighting major activities, events, and issues within each of the stages of policy cycle.

### Problem identification /Agenda setting

#### Historic restrictions leading to unsafe abortion

Nepal has viewed abortion as morally unacceptable and legally punishable since a long time [13]. This prohibition was codified in the Country Code of 1854 and was kept in the 1963 revision. The laws imposed severe penalties, including imprisonment for abortion-related acts [13, 46]. Abortion was only allowed to save a woman’s life, and this required a medical certificate from two physicians. The law did not allow abortion even in the specific conditions like rape, incest, or fetal abnormalities [13, 14, 47, 48].

As a result, women—especially survivors of rape and incest—were left with few options. They could either carry the pregnancies to term or take a risk for an unsafe abortion. Those who choose the second option faced severe complications or even the charges of infanticide [13, 47, 48]. By 2000, Nepal’s maternal mortality ratio (MMR) was 539 per 100,000 live births, which was one of the highest in Asia. At this time, unsafe abortions were linked to over half of the maternal deaths reported in the hospitals [13, 15, 47]. The impact was also faced in the prisons, where one in five women were imprisoned for abortion-related charges [14, 46]. Together, these figures highlight the profound health and social consequences of Nepal’s restrictive abortion laws.

#### Framing unsafe abortion practice as an issue

Until the early 2000s, the debate around abortion policy in Nepal was deeply polarized. On one side, there were pro-choice groups—including health experts, women’s rights groups, Nepal Society of Obstetricians and Gynaecologists (NESOG), Non-governmental Organizations (NGOs), journalists, and legal professionals. They argued that criminalizing abortion forced many women to seek unsafe abortions through clandestine method, which has contributed in higher maternal mortality ratios [46, 49]. They supported legalization with evidence: substantial number of unsafe abortion and imprisonment of women. For them, abortion was both a public health and human rights issue.

Conversely, pro-life groups; made up of conservative civil society members; opposed the reform agendas. They framed abortion as a morally wrong, socially harmful, and warned that it could lead to the risks of sex-selective abortion or be misuses as a form of contraception [46, 49]. Some civil society groups and media outlets went further, claiming that pro-choice advocacy threatened traditional societal values by promoting westernized sexual norms [48]. Despite this opposition, pro-choice coalitions had greater influence, paved the way for legal reform.

When democracy was restored in 1990, it opened the political space for progressive legal changes and acknowledging the ideological pluralism existing in the society. This political shift allowed broader participation of relevant stakeholders, including civil society and advocacy groups. They actively campaigned to lift the abortion bans by presenting strong evidence and lobbying with parliamentarians for legal reforms [13, 15, 16, 46]. At the same time, Nepal’s commitments to international conventions, such as CEDAW, ICPD, and the Beijing Platform of Action, added the momentum for legal reforms [13, 16]. Together these national democratic reforms and international commitment created a conducive climate for reform, empowering policymakers to amend the abortion law in 2002.

#### Mobilizing support for addressing the issue

A broad coalition of pro-choice groups came together to frame unsafe abortion as an urgent policy issue, building momentum for legal reform [46]. This coalition brough together a wide range of players: policy directors (politicians), policy approvers (members of parliament of both houses, and the Parliamentary Committee on Law, Justice, and Parliamentary Affairs). It also brought the advisors such as NESOG members, public health experts, researchers, civil society representatives, journalists, and policy advocates from the Nepal Bar, other professional councils, and women’s rights organizations. Despite their diverse background and interests, they united behind the same cause [13, 46–49].

NESOG pointed out that poorer, less-educated women faced greater barriers to accessing SAS [46]. Public health experts linked Nepal’s high MMR to unsafe abortion practices, documenting traditional dangerous methods like inserting medications, herbs, or foreign objects into the uterus [13, 46]. Legal advocates and women’s rights groups emphasized the injustice faced by women due to existing laws, arguing that they re-victimized them—first through assault and then through prosecution and imprisonment [13, 46]. They also highlighted the difficulties in obtaining citizenship for children born from rape or incest, since the law required proof of the biological father’s identity [13].

Journalists helped to expose wrongful prosecutions by drawing a clear line in their reporting between abortion, miscarriage and infanticide [13, 50, 51]. Human rights advocates revealed systemic inequities, where wealthier women could bypass the law by bribing doctors to obtain false medical reports, while poorer women were imprisoned for the same actions [13, 15, 47]. Together, these advocacy efforts exposed the legal and social injustices faced by women. This in turn helped to shift the public opinion in favour of amending the abortion law in 2002.

### Policy formulation

The key theme in this policy cycle stage was the legislative amendment process. During the 1990s, unsafe abortion was recognized as a major driver of maternal deaths, which generated widespread calls for legal reform [13, 15, 46]. A proposal was made as early as in 1973 to amend the criminal code and allow abortion under specific conditions. But, meaningful legislative reforms occurred only after political changes of 1990s [13, 15].

In 1997, Sunil Kumar Bhandari, a member of the Upper House and the chair of the Family Planning Association of Nepal, registered the Pregnancy Protection Bill as a private member’s bill. His bill aimed to protect wanted pregnancies and legalize abortion under specific conditions, including rape, incest, and threats to the woman’s life [13, 46]. Despite his individual efforts, the bill was rejected by a parliamentary majority [13, 15]. When Bhandari reintroduced the bill in a later parliamentary session, he faced tough questioning and demands for clarification—even from his own party, Nepali Congress [13].

**Fig. 3:**
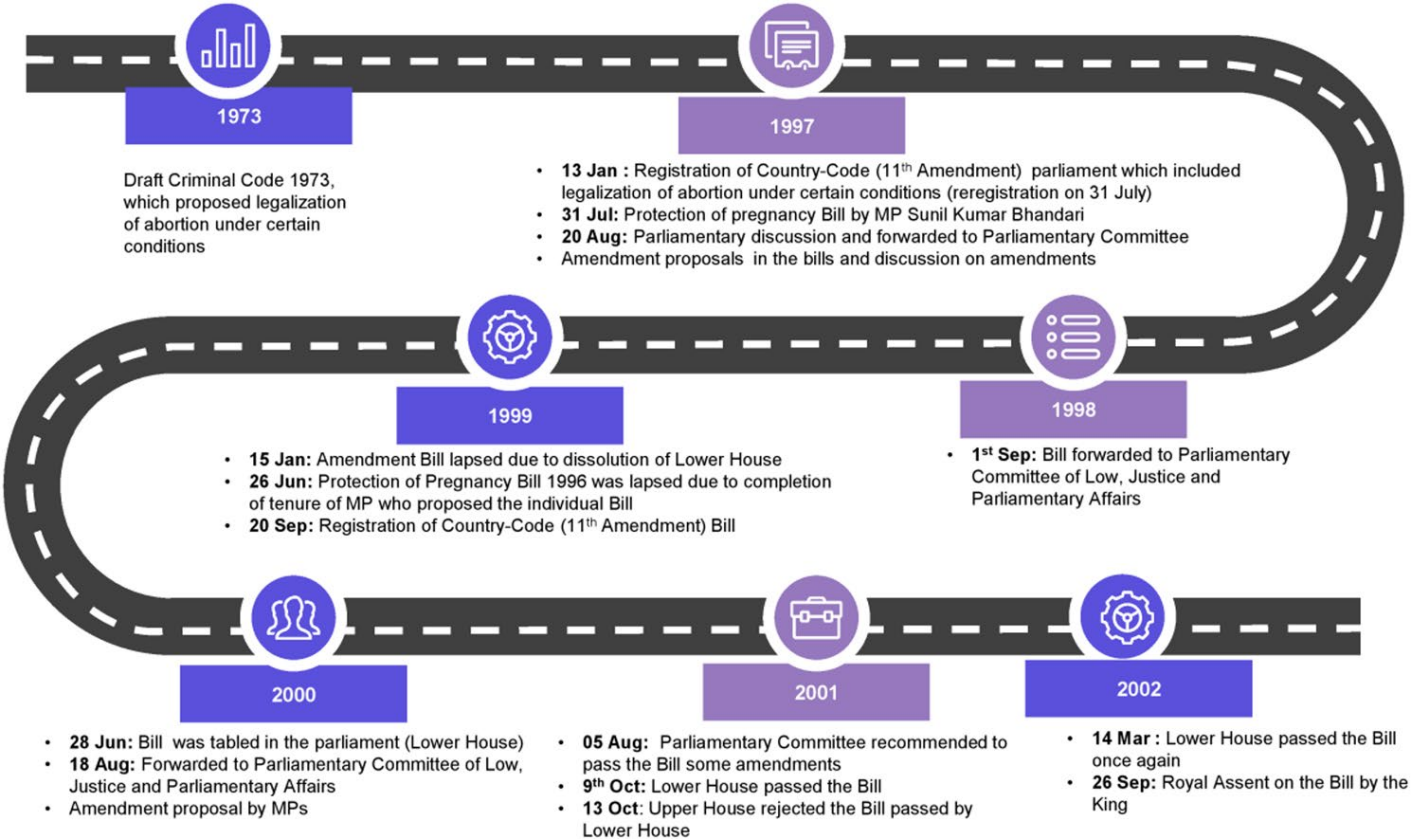
Key policy milestones in Nepal’s path to abortion legalization (Pre-2002)

A pivotal moment came when the government decided to re-register the Country Code (11th Amendment) Bill, building on Bhandari’s earlier proposal and years of advocacy. The bill proposed legalizing abortion under three conditions: (a) within 12 weeks with the husband’s consent for married women, (b) up to 18 weeks in cases of rape or incest, and (c) at any stage if the pregnancy endangered the woman’s life, health, threatened her health or involved risks of severe fetal abnormalities [13, 15, 46]. These legislative efforts marked a critical step toward addressing advancing women’s reproductive rights in Nepal which is shown in Fig. 3.

### Policy decision-making (Adoption)

Key themes in this policy stage include parliamentary amendment proposals, civil society influence and analysis of policy alternatives.

#### Parliamentary amendment proposals

The key aspect of legislative process was deep engagement with the Country Code (11th Amendment) Bill in parliament. The bill went through debates and amendments in committees and beyond [13, 15]. Altogether, 72 amendment proposals were registered by 51 parliamentarians, which reflects how actively the lawmakers were engaged shaping the abortion law [13]. The Parliamentary Committee worked closely with civil society groups and played a crucial role in shaping the bill. For example, the committee pushed to include ban on sex-selective abortion, removed spousal consent requirement to protect women’s autonomy in access to services, and introduced stricter punishments for coercive or forceful abortions [13, 15, 46]. These amendments strengthened the bill, with a clear focus on women’s autonomy and justice.

#### Influence of civil society

Policymakers and civil society organizations carefully evaluated policy alternatives, ranging from keeping the restrictive laws in place to full legalizing abortion. Civil society helped to organize open discussion among parliamentarians and stakeholders, comparing the existing laws with the proposed reforms to show health and rights benefits [13, 15, 46, 48, 49]. This participatory process helped build consensus and helped overcome political and social resistance.

#### Analysis of policy alternatives

The amendment of Country Code Bill was initially approved from the lower house but was subsequently rejected by the upper house. This meant the lawmakers had to go back and reach the consensus on a revised policy [13, 15]. During this phase, policymakers carefully weighted the policy alternatives. They conducted health and social impact assessments and held broad consultations with stakeholders. Their goal was to compare the existing restrictive law against the potential benefits of legalization. Importantly, they reached to a consensus to prohibit sex-selective abortion in the proposed bill [13, 15, 46].

After the law was passed in 2002, the government and non-governmental organizations launched public awareness campaigns. They used mass media platforms such as radio, television, newspapers alongside community workshops, street dramas, and door-to-door outreach to inform people about the new law and available services. Simultaneously, healthcare providers received training on how to deliver the services, make referrals, recording and reporting. The training also covered critical issues like maintaining privacy and confidentiality [12, 13, 16].

### Implementation of safe abortion policy

Key themes in this stage include legislative reforms, capacity building, phased service rollout, and judicial orders to ensure equitable access to SAS.

#### Legislative reforms

Procedural legislative reforms were equally important as the law itself for effective implementation. The MoHP institutionalized SAS by endorsing key documents, including Safe Abortion Policy (2003), procedural orders, strategic plans, implementation plans, and training and reference manuals [12, 16, 48, 52]. These procedural policy documents ensure nationwide access to free, affordable, and quality SAS by mandating three core standards: (a) availability of skilled providers, (b) adequate medical equipment and essential drugs, and (c) adherence to established clinical protocols. They also focused on training health workers and strengthening referral systems to manage complications effectively [52].

By partnering with private clinics and NGO-run health facilities, the government was able to expand the services further up to the rural areas [16, 52]. To standardize and expand the services, the government introduced several plans including Medical Abortion Scale Up Strategy (2008), National Safe Abortion Service Implementation Guidelines (2011), and Medical Abortion Drug and Equipment Supply Guideline (2013) [12].

Later, the new constitution of 2015 recognized reproductive health rights as fundamental rights [53]. This commitment was reinforced by two major laws: Safe Motherhood and Reproductive Health Rights (SMRHR) Act and its regulation [54, 55] and Public Health Service Act [56]. Building on this, the government started providing free basic healthcare including SAS at public health facilities from 2016 onwards [57].

**Fig. 4:**
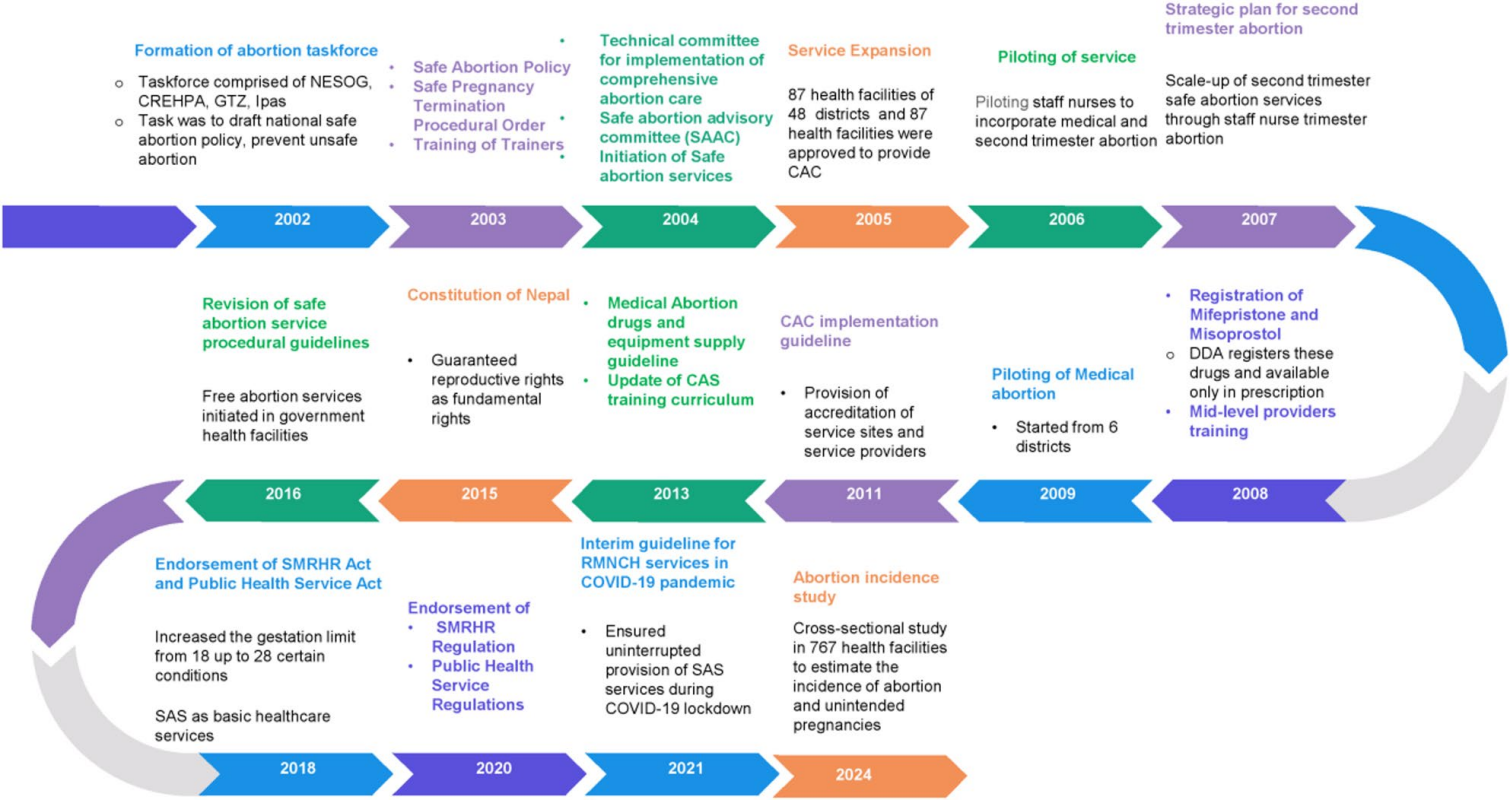
Evolution of safe abortion policy implementation in Nepal since legalization in 2002

The Safe Abortion Service Program Management Guideline of 2021 delegated more power to local governments. Under the new federal system, the provincial and local governments can now approve services sites and service providers within their areas [57]. As a result, between 2021 and 2022, 136 new sites and 300 new providers were approved, with an additional 7 hospitals and 121 providers added in fiscal year 2023/24 [32, 58]. As Fig. 4 shows, this has made the difference in reaching women to rural areas [32, 57].

#### Capacity building

To build a strong pool of service providers, the National Health Training Centre (NHTC) rolled out a cascade training programme with technical support from Ipas. They started by training 20 master trainers, who then conducted Training of Trainers (ToT) programmes across the country. The NHTC also updated its training curriculum to align with the 2011 Comprehensive Abortion Care (CAC) Guidelines and WHO’s Safe Abortion Technical Guidance.

By 2025, Nepal’s SAS network has grown significantly. It comprised 1,685 MA sites, 730 MA/MVA sites (≤ 10 weeks), 350 MVA sites (≤ 12 weeks), and 63 dilation and evacuation (D&E) sites, supported by 4,254 MA providers, 1,422 MA/MVA providers, 2,859 MVA providers, and 187 D&E providers [59].

#### Phased rollout

The SAS was rolled out in a well-structured, phased approach to ensure systematic expansion. Services began in 2004 at Paropakar Maternity and Women’s Hospital in Kathmandu [15, 60]. Within one year, the services were expanded up to 87 health facilities (including 53 government and 34 non-governmental facilities) across 48 districts. This early expansion established a nationwide network for CAC, prioritizing MVA services [16, 60]. These services initially focused on central hospitals and gradually extended to district hospitals and primary health centres, thereby increasing accessibility across the country [16, 19].

From 2006 to 2009, the programme focused on decentralisation and diversification to improve access. During this period, the government allowed staff nurses to provide services and piloted medial abortion (MA) for the first trimester in six districts. Later, MA services were expanded to health posts with birthing centres. This helped reach remote communities where second-trimester abortion services were less feasible [4, 12].

By 2014, second-trimester abortion services were available in 22 hospitals, supported by 46 trained service providers [18, 61]. Training for second-trimester abortion was suspended in 2015 due to concerns about sex-selective abortions and was later resumed after two years [61].

Despite these advancements, service providers faced major challenges: many women delayed seeking care until around 18 weeks of gestation, often due to multiple barriers. To address this, NESOG pushed to extend the legal gestational limit for second-trimester abortions. This advocacy agenda was addressed through SMRHR Act, which extended the gestational limit to 28 weeks for pregnancies from rape or incest from the previous 18 weeks limit [55].

#### Judicial order and equitable access

Although abortion services had been available since 2004, they remained out of reach for many poor and marginalized women. A major turning point came in 2009 with the Supreme Court’s ruling in the case of *Laxmi Devi v. Government of Nepal*. The verdict expanded reproductive health rights to explicitly include abortion, childbirth and access to safe and affordable abortion services [46, 49]. The court made it clear that financial barriers should not prevent women from accessing care, and directed the government to ensure services are available, accessible, and affordable for all women [46, 49]. This landmark ruling helped to expand the services up to the rural areas, reducing inequality in service access and acknowledge reproductive rights as fundamental rights of the 2015 constitution.

### Policy evaluation

Key themes in this policy stage include trends in service utilization, changes in the sex ratio at birth (SRB) following abortion legalization, evidence of abortion as a family planning tool, the impact of SAS on maternal health, and inequalities in access to services.

#### Types and trend of service utilization

**Fig. 5:**
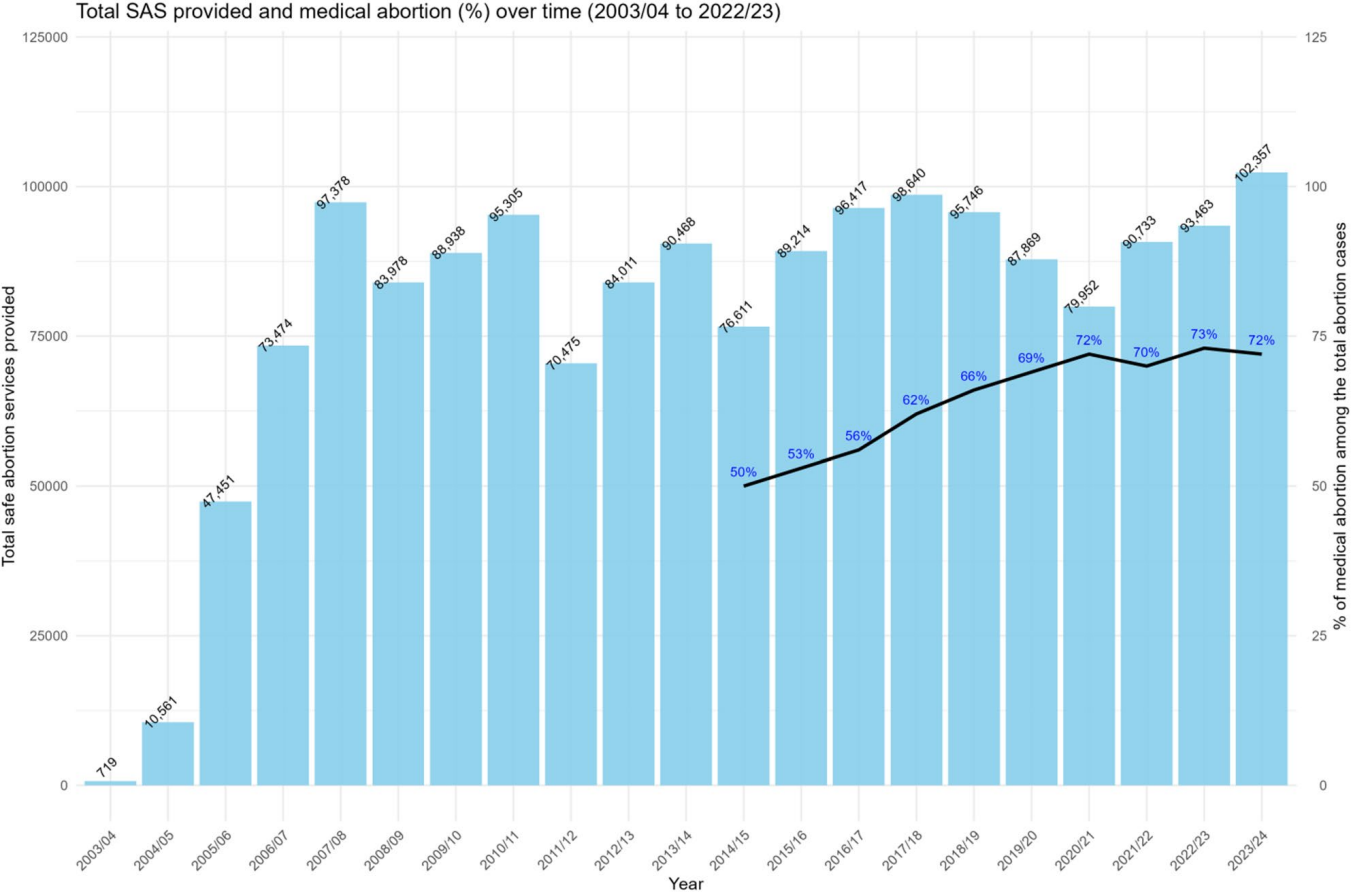
Annual safe abortion service (SAS) cases and medical abortion share in Nepal Post-legalization. Data source: authors’ calculation based on the information from [16, 20], 33– [40]

Following the legalization in 2002, Nepal witnessed a rapid increase in the utilization of SAS. Since 2003, over 1.6 million women have accessed these services [16, 20, 33–40]. Between fiscal years 2014/15 and 2023/24, there was a notable shift in the abortion methods women used. The share of MA increased from 50% to 73% of the total SAS. Yet, despite this change in service preference, the total number of abortions each year have stayed steady at approximately 100,000. This trend reflects a growing preference for medical methods over surgical alternatives, which is driven by several factors such as convenience, accessibility, and safety (Fig. 5).

#### Unmet need for family planning and reliance on abortion

According to the 2022 DHS survey, 21% of women of reproductive age in Nepal continue to experience unmet need for family planning (13% for limiting and 7% for spacing) [62]. There is a strong indication that abortion is being used as a means of family planning in Nepal. Studies consistently show that over half of all pregnancies are unintended [22, 63]. Research also shows that more than 62% of such unintended pregnancies end in abortion. This highlights how crucial is SAS in helping women managing reproductive choices [63]. This high figure also suggests that abortion functions as a substitute for family planning methods, indicating limited access to contraceptive services [64].

#### Impact on maternal health and service delivery

The maternal mortality ratio has dropped sharply—from 539 per 100,000 live births in 1996 to 151 per 100,000 live births by 2021 [20]. Several factors have contributed to this decline. Key policy interventions such as the National Safe Motherhood Policy, Skilled Birth Attendant Policy, Maternity Incentive Program, and the Safe Abortion Policy have played critical roles in shaping this maternal health outcomes [20, 40]. At the same time, improvements within the health system—like better access to healthcare and higher service quality—helped ensure that women receive timely and appropriate support. Broader societal changes also made a difference: rising levels of maternal education, greater purchasing power and access to cash due to remittance-based economy, falling fertility rates, and growing community awareness all have contributed to better maternal outcomes [20, 40].

The safe abortion policy was designed to reduce high rate of maternal deaths by making safe and legal abortion services available. Since legalization, the share of maternal deaths linked to abortion has dropped significantly. Unsafe abortion, which once accounted for nearly 20% of all pregnancy-related maternal deaths in hospitals [16] has now declined to 5% as of 2021 [65]. While SAS are widely recognized as an important contributor to improving maternal health, the exact scale of their impact on reducing maternal mortality has not been fully studied [17, 66].

However, despite this progress, important gaps remain in fully achieving the policy’s objectives. Awareness among women of reproductive age remains low; nearly two decades after the legalization, only 41% of women know the legal status of abortion [67]. This lack of awareness has limited women’s ability to seek and access safe services. In addition, service provision remains fragmented: only 48% of abortions are performed at authorized sites by trained providers. This means over half of abortion procedures take place in unauthorized settings [22].

#### Inequality in access to services

Despite noteworthy progress in expanding SAS since legalization, substantial inequality in access remains. This is especially true for low-income women and those residing in remote mountainous regions [17]. A recent study estimated that approximately 333,000 abortions occur annually in Nepal; that’s 3.67 times higher than the 90,733 officially recorded cases [20, 22]. This wide gap suggests that many women rely on self-managed MA drugs or unsafe traditional methods outside the formal healthcare channel [28, 68]. Evidence from recent DHS survey and other studies indicates that these barriers contribute to the unintended pregnancy rate exceeding 53% within this population [17]. Moreover, over half of all abortions still occur outside the formal healthcare system, often involving unregulated providers such as pharmacies and medical shops. This reliance on informal providers exposed women to significant health risks, undermining the safety and quality of abortion care [22, 63].

The weak regulation of MA drugs in Nepal is further undermining equitable access to SAS and weakening the impact of the safe abortion policy. Although the government restricts MA brands (combined regimen of mifepristone and misoprostol) to prescription-only distribution through accredited providers [28], more than 17 registered and unregistered brands are available in the market. This is often due to cross-border leakage and comparatively cheaper prices [28, 68]. This regulatory gap allows private clinics and pharmacies to charge unregulated fees, which in turns blocks equitable access to services [67]. The widespread availability of unregistered MA brands raises serious safety concerns, including poor drug quality and inadequate counselling from non-accredited providers. Furthermore, unregulated fees in private facilities create financial barriers, particularly for rural and low-income women. While over 70% of MA cases are recorded in the formal system, widespread underreporting suggests even greater levels of informal use [20, 39, 40].

Even with constitutional guarantees since 2015, heavy reliance on MA provided by auxiliary nurse midwives and staffs nurses for pregnancy up to 10 weeks shows that barriers to accessing surgical methods like MVA still exist [18, 32, 67]. Rural and low-income women disproportionately face challenges accessing surgical methods due to high costs, transportation difficulties and limited service availability [18]. For example, second-trimester services require specialized and trained providers like obstetrician/ gynaecologists or MDGP doctors with postgraduate training; yet in 2021, only 34 of 1,516 facilities were offering second-trimester services, and nearly all of these facilities are concentrated in urban areas [32, 67].

#### Impact in sex ratio at birth and indications of sex-selective abortion

**Fig. 6:**
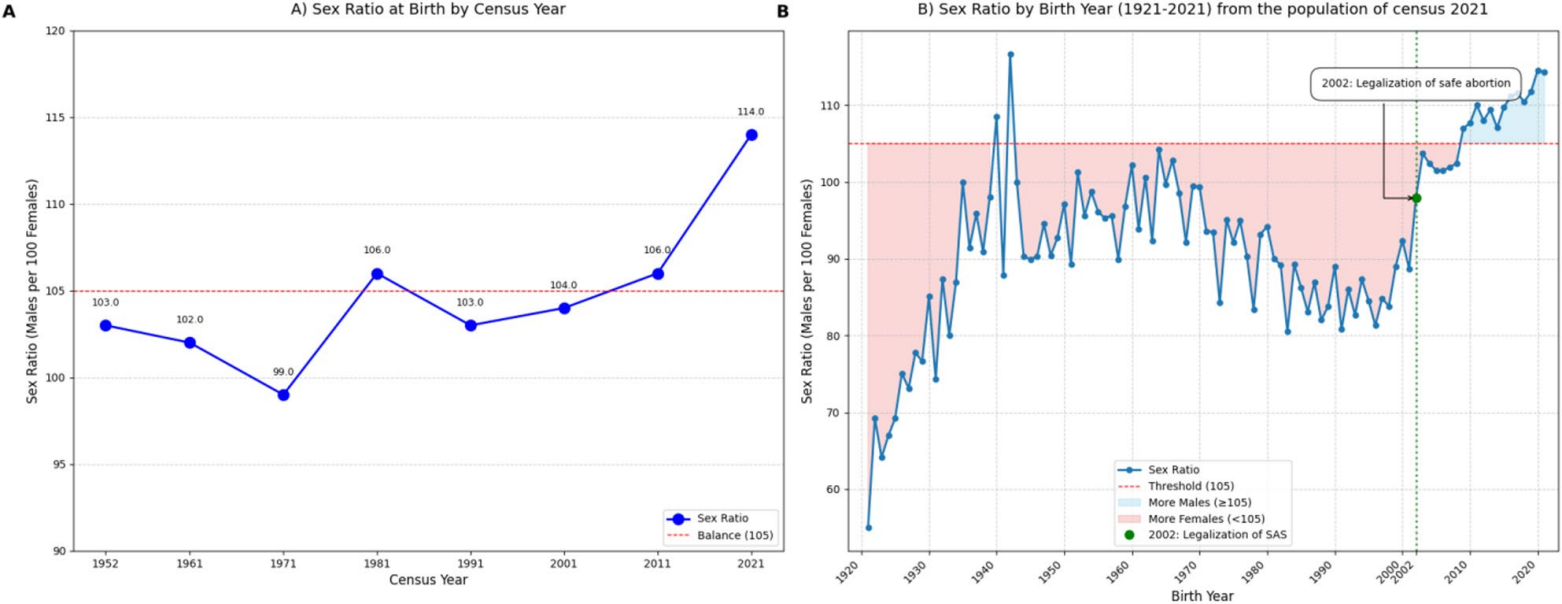
Average sex-ratio by birth decade and sex ratio by birth year. Data source: Panel **A** [41] and Panel **B** [42]

The legalization of abortion coincided with a notable shift in sex ratio at birth (SRB), characterized by an increasing proportion of male births relative to female births recorded annually since 2003 (Fig. 6).

Panel A, which shows the SRB since 1952 national census illustrates an upward trend over time [41]. Similarly, Panel B, based on 2021 census data confirms this trend. It shows a consistent rise in male births compared to females following legalization [42].

Multiple studies have raised concerns regarding this growing imbalance in Nepal’s SRB. Analyses of demographic and health survey (DHS) data [69], DHS and census data [70], and DHS combined with hospital-based data [71] have documented evidence of skewed sex ratios favouring males. Media reports have also highlighted these trends, drawing attention to the social and ethical implications of rising SRB in Nepal [72–77].

There are obvious signs that sex-selective abortions in Nepal have been increasing over time. This is further exacerbated by deep-rooted social norms that favour sons over daughters. As a result, the SRB has increasing skewed, which widens the gender inequality. This practice not only violates medical ethics but also carries serious social consequences that reinforces gender inequality in the society [69, 70, 78, 79].

At the same time, the fertility rate in Nepal has dropped sharply. The total fertility rate (TFR) fell from 3.25 in 2001 to 1.9 in 2021, which is below the level to replace the population [80]. While the exact relationship between sex-selective abortion and falling fertility has not been extensively studied, this trend may reflect the influence of son preference. Couples may be using sex-selective abortions to ensure that they have a son while also choosing to have fewer children.

## Discussion

This review synthesized how abortion policy in Nepal was conceptualized and developed over the period and the challenges it faced along the way of implementation. Initially, back in 1970s, the agenda for SAS was driven by international actors, particularly the United States, who saw it mainly to control population growth. Over time, the agenda shifted. Global events like ICPD and Beijing conferences helped to change the narrative, pushing towards rights-based approach. The agenda was led by NGOs and INGOs, who framed the agenda of access to abortion within the broader principles of reproductive justice and women’s autonomy. This shift played a critical role in setting the agenda successfully and formulating progressive legislation in Nepal.

Even though Nepal’s abortion laws have gradually improved over time, there is a clear gap between the policy and practice, revealing a clear paradox which cannot be ignored. While abortion is guaranteed through the constitution, women in rural and remote areas still struggle to access the services due to the uneven distribution of service-delivery sites. On top of that, there is lack of comprehensive data on whether the policy has reduced maternal morbidity and mortality from unsafe abortion, making it difficult to assess the effectiveness of the of policy. Moreover, there is evidence of unintended consequences, like sex-selective abortion, which show up in a raising SRB, and more women are using abortion as a means of birth control, seen in higher rates of repeat abortions. This contradiction between progressive policy and its practical outcomes suggests that while the SAS policy in Nepal has been a powerful advocacy tool for advancing women’s rights rhetorically, in practice it has fallen short. It has not guaranteed fair access to quality care for all women, which means it has not been able to fulfil the commitments of protecting women’s reproductive rights as a fundamental human right.

### Democracy opens the door for progressive abortion law

Nepal’s safe abortion policy is widely regarded as a success in agenda setting and legislative advancement for reproductive rights. The groundwork for reform began back in the 1970s, when US funded abortion-related research and seminars were conducted. This led Nepal Law Commission to purpose a new Criminal Code in 1973, that aimed to liberalize abortion as a family planning method [13, 15]. However, these reform efforts were interrupted after Reagan Administration introduced Mexico City Policy, which cut-off US funding to any organizations that used abortion as one of the methods of family planning [81]. Since the US was the main donor for health and family planning in Nepal at that time, this political changes in the US hindered the progress on abortion law reform in Nepal [15, 81].

Things shifted again after democracy was restored in 1990s. The democratic system of governance opened the door for more public participation and gave rise to NGOs and INGOs advocating for women’s rights and reproductive health. This shifted the discourse from abortion as a family planning method to broader reproductive rights and women’s autonomy [13, 15].This ideological shift, along with democratic reforms, created a more favourable environment for policy change. For example, parliamentarians like Sunil Kumar Bhandari, who got the opportunity to participate in ICPD later introduced Pregnancy Protection Bill in the parliament as a private bill [13, 15]. Public health concerns over unsafe abortion, the rise of media after the 1990s political changes, and court rulings that supported gender equality all paved the way for Nepal to finally liberalize its of abortion law in 2002 [13, 15].

International NGOs have played a pivotal role in making safe abortion services a reality in Nepal and helped to put the policy forward to practice. Organizations like DFID-funded Nepal Health Sector Support Program, Marie Stopes International, Family Planning Association of Nepal, Forum for Women’s Law and Development, Safe Motherhood National Federation, PSI, CREHPA and Ipas have been the key players in policy implementation. They supported to expand the services, helped to draft key procedural legislation like Safe abortion policy and Safe Pregnancy Termination Procedural Order, and worked to strengthen the health system. These organizations also trained healthcare providers, supplied essential equipment and medicines, and improved the health information system to so services could be delivered more effectively [13, 46, 48, 82].

The 2015 constitution guaranteed reproductive health rights as fundamental rights. At the same time, the new federal system after 2015 constitution gave provincial and local governments the power to expand service sites and approve providers within their own areas. This shows that a democratic system and an open society—one that accepts pluralism—are essential for progressive abortion laws.

### Repeated abortion practice as a means of family planning

The unmet need for family planning has decreased from 28% 2001 to 21% in 2022 [62, 83] which is a remarkable progress, but it also shows considerable gaps in both the coverage and effectiveness of family planning programs. These gaps are largely driven by limited access to contraceptives and failures in method use [22, 63].

Similarly, more unintended pregnancies are ending in abortion, including repeated abortion, which indicates a systemic challenge in family planning program. In 2016, about 62% of unintended pregnancies ended in abortion [63]. When we looked at a more recent similar study by Ghimire and colleagues [22] that figure has risen to 72.6%. (It’s worth noting that the recent study didn’t specifically highlight this figure the way the 2016 one did.) This 10-percentage-point increase since 2016 suggests that instead of improving the family planning coverage, we’re seeing a growing reliance on abortion to manage unintended pregnancies.

### Safe abortion on paper, but still unsafe in practice

After the legalization of abortion, there has been a significant reduction in maternal deaths. This can be attributable to decrease in severe abortion-related complications [82]. However, despite this progress, there exists persistent challenges. A major problem is the lack of comprehensive data on how SAS impact deaths related to abortion, which have made difficulty in evaluating the policy’s effectiveness. Second, many women are still unaware of the service availability, which prohibits them from seeking the abortion care when they needed. The reality is that unsafe abortion services persist, even the policy intends to eliminate it. The reduction in abortion-related deaths from 20% to 5% is a major achievement after the implementation of the policy. However, two issues remain: low awareness of SAS and continuous existence of unsafe abortion. Together, these issues highlight the gap between what the policy intends and what is happening.

Clandestine abortion can lead to several physical and psychological health consequences for women. Physical complications include infections, haemorrhage, and, in severe cases, potentially life-threatening conditions. Abortion-related obstetric issues, such as preterm birth, low birth weight in subsequent pregnancies, ectopic pregnancy, and infertility, have also been documented [2]. Psychologically, women may experience depression, anxiety, or post-traumatic stress disorder (PTSD) following an abortion [2]. Additionally, abortion imposes a financial burden on individuals and the health system, particularly when complications require additional medical care [1, 84]. These multifaceted health and economic challenges highlight the urgent need to strengthen family planning programs to reduce unintended pregnancies and abortion reliance while ensuring equitable access to SAS.

### Promises unmet, poor access and inequity

Even though Nepal’s constitution guarantees safe abortion as a right, many women, especially those who are poor, come from rural and marginalized families still cannot access to SAS. The barriers include lack of awareness regarding the services [25, 29, 85, 86], inadequate knowledge about where to find the service providers stigma, discrimination, and negative attitude of service providers [4, 67]. Other structural challenges such as distance, transportation cost, and lack of privacy, bureaucratic hurdles like spousal consent requirements and supply side barriers such as services interruptions also contribute in widening the inequality [4, 25, 28, 67]. This has created a paradox: Nepal has one of the most progressive abortion policies South Asia [87], however, many women are unable to exercise their constitutionally guaranteed rights. These systemic shortcomings directly undermine the country’s commitment to reproductive rights in both policy and practice.

Despite substantial achievements, many women still don’t know about the services or their rights, stigma around abortion remains strong. In addition, there aren’t enough trained providers or facilities, the health infrastructure is inadequate, and opportunities for training and capacity building are limited [22, 26, 67, 82]. These ongoing challenges highlight a major gap between policy commitment and practice on reality. Even after two decades after legalization, Nepal is still struggling to fully achieve the goals of safe abortion policy.

### A silent crisis of unseen sex-selective abortion

Sex-selective abortion has dire consequences. It can lead to a smaller number of women, increased violence against women, and long-term effects in gender equity and social stability. By allowing the preference of son in the society by overriding the medical ethics, it reinforces the idea that girls are less valuable, leading to societal discrimination, constrains reproductive choices and challenge both public health and social justice. Empirical data from other countries show additional social impact. In China, the gender imbalance because of sex-selective abortions have created profound difficulties for uneducated and economically disadvantaged men in finding their life partners. This has caused adverse psychosocial outcomes such as reduced self-esteem, increased psychological vulnerability, and higher rates of antisocial behaviour [88].

Sex-selective abortions break a fundamental rule of medical ethics. It places a cultural preference for a son over the fundamental principles that all people are equal. This unethical practice has grave consequences. First, it reinforces deep-rooted patriarchal norms that teaches society to value males more than females [89]. Second, it leads to systemic discrimination against women and girls. Third, by prioritizing male births, it further marginalizes females in the family and society.

Addressing this complex issue requires working in two areas: raising awareness in the community and financial support for families to raise daughters. First, educational campaigns aimed at raising awareness about the social values of girls and promoting gender equality can teach the communities that girls are equally valuable as boys. Second, economic incentives, such as scholarships and conditional cash transfers for families with daughters can help reduce the economic burden associated with raising daughters in the family [90]. When girls are supported through school scholarships, and vocational training, it not only empowers them but also contributes in changing the traditional societal attitudes that discriminates daughters [69].

Likewise, strong regulations are essential to stop sex-selective abortion by ensuring healthcare providers follow the ethical standards [91]. This requires effective enforcement mechanisms, strong monitoring system, and accountable service providers. The new 2025 National Population Policy of Nepal recognizes this need [92], however, implementing and enforcing these measures remains difficult without efficient structural mechanisms and adequate resources.

### Policy implications

Even though Nepal’s constitution guarantees reproductive health rights that includes right to SAS, many women still do not have access to SAS, and sex-selective abortions are on the rise. This shows the government need to step in with targeted policy interventions to ensure equitable reproductive healthcare. Currently, more than half of abortions occur outside the formal healthcare system, often through unregulated providers. The rural and low-income women are affected much due to this inequality. They face significant barriers to safe services because accessing SAS are difficult for them due to limited availability or they don’t have access to quality care. Consequently, many have no choice other than performing unsafe or unregulated options, putting themselves to serious health risks.

Nepal’s transition to federal system have created an opportunity for local governments to develop the policies and strategies that fit their own community’s need. Nevertheless, the high number of unplanned pregnancies—many of which end in abortion—indicates major gaps in family planning program. Addressing this requires range of efforts including expanding access to contraceptive services and reducing the unmet need for family planning.

The fact that Nepal’s SRB remains skewed indicates that sex-selective abortion is still happening, even though it is against the law. To combat this, Nepal must strengthen regulatory mechanisms to prohibit illegal sex determination for abortion and enforce penalties for those who breaks this rule. Furthermore, discrepancies between reported and estimated abortion cases indicates serious flaws in existing data management system. Without strong data management system, it is difficult to track what is really going on. Strengthening data management system is therefore essential for accurate monitoring, policy formulation, and resource allocation. Fixing these issues through focused policy efforts is key if Nepal wants to live up to its constitutional promise of protecting reproductive rights for all women.

### Study limitations

The strength if this paper is it uses a comprehensive mixed-method approach for policy analysis and drew on a wide range of data sources. However, there are some limitations that need to be considered. First, the literature search was purposeful rather than systematic, which means that there can be possibility of selection bias. Even so, the review brings together the available literature in the field and provides a future perspective in the abortion policy and practice landscape in Nepal. Our findings highlight several issues that calls for further research to evaluate the implementation of safe abortion policy of Nepal through different dimensions.

Second, this study only uses secondary data and does not include primary information on maternal deaths related to abortion. Without this first-hand data, it was difficult to fully evaluate how the safe abortion policy has affected on maternal health outcomes. Gathering primary data would provide clear picture of how the policy has influenced abortion-related mortality and morbidity on the ground.

Finally, while the study points out sex-selective abortion is happening and links these practices to son preference, it does not offer detailed quantitative analysis of the magnitude of the issue. To truly understand the impact of sex-selective abortion on fertility patterns in Nepal, we need large-scale quantitative studies that can measure the issue more precisely.

## Conclusions

Nepal’s transition from the criminalization of abortion to recognizing it as a as a fundamental reproductive right in constitution is a historic achievement. This major shift was driven by years of advocacy, a landmark legal reform in 2002, and ongoing progressive policies. Together, these efforts have expanded SAS and improved access by decentralizing the care and allowing more types of health workers to provide services.

Yet, despite these advances, major gaps remain—especially for rural, low-income, and marginalized women. They still face barriers like uneven access to services, stigma, and simply not knowing where to go and whom to go for seeking the services. A major concern is that over half of all abortions still occur outside the formal healthcare system, often through unregulated providers, which puts women at serious health risks. Even though the constitution guarantees safe abortion, for many women that rights are just on papers rather than the real world. Furthermore, using abortion as a substitute for family planning and ongoing problem of skewed SRB raises deep ethical and societal challenges. These trends reflect gender discrimination and threaten long-term social stability.

To address these issues, Nepal needs to focus on few key reforms. First, it must invest more in training healthcare providers, making sure it can expand equitable access, particularly for second-trimester services. Second, local governments need to step up and enforce the existing rules to curb sex-selective abortions, controlling the sale of unauthorized abortion drugs, and ensuring that the service cost is fair and clear. Third, public awareness campaigns should target to address misinformation and let women in the underserved communities know their legal rights and the places to seek the services. Fourth, making the contraceptives more widely available can help to reduce the number of unplanned pregnancies and ultimately the need for abortion services. Finally, a comprehensive approach—combining education, stricter enforcement, and economic incentives for daughters—is essential to tackle the root causes of sex-selective abortion and uplift the status of girls. If Nepal follows through on these reforms, it can turn its constitutional promises into real, fair access for all women.

## Data Availability

All data generated or analysed during this study are included in this published article.

## Abbreviations

CAC: Comprehensive Abortion Care
CEDAW: Convention on the Elimination of All Forms of Discrimination against Women
CREHPA: Centre for Research on Environment Health and Population Activities
D&E: Dilation and Evacuation
DFID: Department for International Development
DHS: Demographic and Health Survey
DoHS: Department of Health Services
FP: Family Planning
ICPD: International Conference on Population and Development
INGOs: International Non-Governmental Organizations
LMICs: Low- and Middle-Income Countries
MA: Medical Abortion
MDGP: Doctor of Medicine in General Practice
MMR: Maternal Mortality Ratio
MoHP: Ministry of Health and Population
MVA: Manual Vacuum Aspiration
NESOG: Nepal Society of Obstetricians and Gynaecologists
NGO: Non-Governmental Organization
NHTC: National Health Training Centre
PSI: Population Services International
PTSD: Post-Traumatic Stress Disorder
SAS: Safe Abortion Services
SMRHR: Safe Motherhood and Reproductive Health Rights
SRB: Sex Ratio at Birth
TFR: Total Fertility Rate
ToT: Training of Trainers
UNFPA: United Nations Population Fund
WHO: World Health Organization
